# Exploring the etiology of colitis: insights from gut microbiota research

**DOI:** 10.1080/19490976.2025.2512010

**Published:** 2025-06-02

**Authors:** Guanru Qian, Xiaolin Chen, Gang Liu, Jiankang Yu, Sijia Zhong, Jiaxi Yang, Yefei Sun, Jianping Zhou

**Affiliations:** a Department of Gastrointestinal Surgery, The First Hospital of China Medical University, Shenyang, Liaoning, China; b Shenyang Medical Nutrition Clinical Medical Research Center, Shenyang, Liaoning, China; c Department of Operation Room, The First Hospital of China Medical University, Shenyang, Liaoning, China

**Keywords:** Colitis, gut microbiome, etiology, dysbiosis

## Abstract

Colitis represents a significant global health concern, with its incidence rising annually in recent years. Despite the unclear specifics of its pathogenic mechanisms, dysbiosis of the intestinal microbiota is recognized as a major factor contributing to various forms of colitis. This article examines the mechanisms through which alterations in intestinal microbiota contribute to the pathogenesis of several common types of colitis. These mechanisms predominantly involve disruption of barrier function, aberrant immune responses, and damage induced by microbial metabolites. This review offers insights that enhance the understanding of colitis pathogenesis.

## GRAPHICAL ABSTRACT

**Figure:**
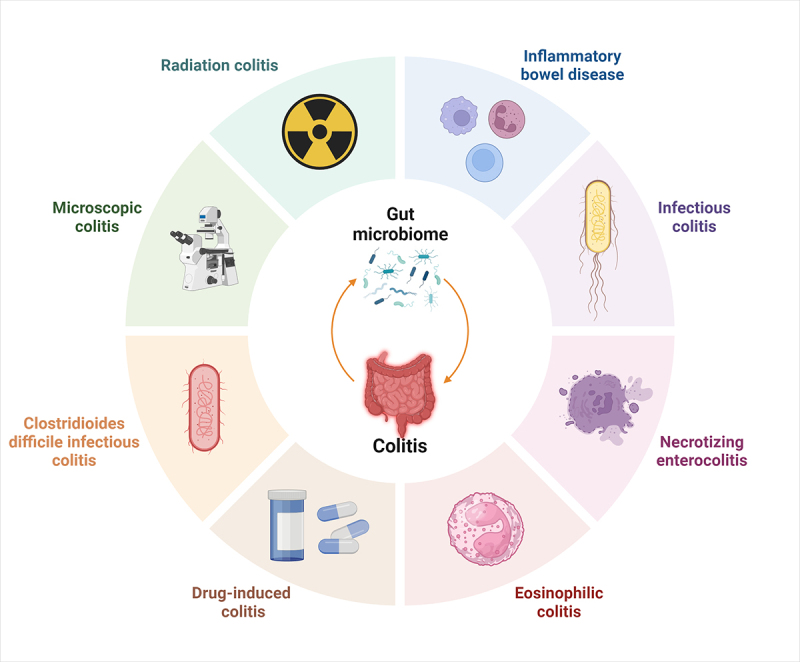

## Introduction

Colitis, a condition of increasing global concern, is characterized by acute or chronic inflammation of the intestines, presenting with symptoms such as abdominal cramps and pain, diarrhea, and hematochezia. This term encompasses a broad spectrum of diseases arising from various etiological mechanisms. Infectious colitis can result from pathogenic invasion, while immune dysregulation may lead to ulcerative colitis (UC) and Crohn’s disease (CD). Additionally, colitis can be induced by exposure to specific drugs or radiation, leading to drug-induced or radiation colitis (RC), respectively. Moreover, various forms of colitis, including necrotizing enterocolitis (NEC), eosinophilic colitis (EC), and microscopic colitis (MC), have multifactorial etiologies. The global prevalence of UC is estimated to be approximately 5 million, with a rapidly increasing incidence and hospitalization rates in countries such as India, China, and Latin America.^1,2^ The diverse pathogenic mechanisms underlying colitis and the extensive patient population necessitate a comprehensive summary of existing reports that focus on the etiology of various forms of colitis.

Among the etiologies contributing to colitis, intestinal dysbiosis, disruption of intestinal barrier function, and uncontrolled inflammation represent common underlying factors across various forms of colitis. In both UC and CD, the interaction between the microbiota and the host plays a pivotal role in disease progression. The dysregulation of the intestinal microbiota, the production of associated metabolites, damage to the intestinal barrier, and specific fungal infections significantly influence the onset and development of UC and CD. In infectious colitis, the activation of the PI3K-AKT pathway and related inflammatory pathways, along with the impact on intestinal epithelial cells, leads to a decreased abundance of probiotics such as *Lactobacillus, Akkermansia, Bifidobacterium longum, Muribaculaceae, Blautia, Butyricicoccus*, and *Roseburia*. These factors are essential mechanisms underlying the progression of infectious colitis. In NEC, the intestinal microbiota plays a key role through the regulation of metabolite production, intestinal barrier integrity, and the expression of pro-inflammatory genes. Similarly, intestinal microbiota-induced allergy exacerbates the infiltration of eosinophils in the intestinal wall of EC. Although ionizing radiation is the direct cause of RC, the development of this condition is further exacerbated by the impairment of the intestinal microbiota. Chronic use of medications, including antibiotics, nonsteroidal anti-inflammatory drugs (NSAIDs), antitumor drugs, and mycophenolate mofetil (MMF), may lead to drug-induced colitis. During this process, the intestinal microbiota may be damaged, thereby affecting drug metabolism and the host’s immune responses. As a rare cause of chronic diarrhea, the pathogenesis of MC is also accompanied by dysregulation of the intestinal microbiota.

Current research on the relationship between gut microbiota and colitis still presents significant limitations and controversies. The primary challenges lie in the restricted ability to infer causality, as most cross-sectional studies cannot distinguish the temporal sequence between microbiota dysbiosis and disease onset, and animal models (such as DSS-induced colitis) are difficult to fully replicate the heterogeneity of human diseases.^3^ Methodological heterogeneity (e.g., sequencing technologies and sampling site differences) and insufficient sample sizes further limit the generalizability of the conclusions.^4,5^ The controversy focuses on the duality of specific microbiota functions (such as contradictory evidence of *Bacteroides* exhibiting pro-inflammatory or protective effects in some studies), the divergence in the efficacy of intervention strategies (the fluctuation in the response rate of fecal microbiota transplantation between 30% and 50%, and the dependence of probiotic efficacy on the host’s baseline microbiota), and the ambiguity in mechanistic understanding (the specific concentration threshold of short-chain fatty acids’ anti-inflammatory effects, and the unclear molecular pathways of microbiota-Treg/Th17 axis regulation).^6,7^ Additionally, the controversy surrounding the concept of “core pathogenic microbiota” and the lack of standardized dietary intervention protocols both highlight the complexity of disease mechanisms and the individualized nature of host–microbiota interactions. In the future, it is essential to integrate multi-omics data through multicenter longitudinal cohorts, develop highly biomimetic disease models, establish standardized procedures for microbiome analysis, and explore precision treatment strategies based on baseline microbiota characteristics, in order to systematically elucidate the dynamic regulatory network of microbiota at the onset and progression of colitis.

In the present review, with an emphasis on intestinal dysbacteriosis, we discuss and summarize the etiology of different forms of colitis including UC, CD, NEC, EC, MC, drug-induced colitis (DC), RC and infectious colitis (IC). We hope our review could bring novel insights for understanding the mechanism of colitis, provide instructions for early screening and detection of colitis and uncover targets for therapy development of colitis.

## Non-infectious colitis

### Inflammatory bowel disease (IBD)

IBD, encompassing ulcerative colitis (UC) and Crohn’s disease (CD), is a chronic inflammatory disorder of the gastrointestinal tract with rising global incidence. While UC primarily affects the colon, CD may involve any segment from the oral cavity to the perianal region. The pathogenesis of IBD involves complex interactions between genetic predisposition (e.g., NOD2 polymorphisms) and environmental triggers (e.g., diet and antibiotics).^2,8^ Emerging evidence suggests that gut microbiota dysbiosis serves as a unifying mechanism bridging these risk factors, offering new perspectives for diagnosis and therapeutic interventions such as fecal microbiota transplantation (FMT) and probiotics (Table 1).

**Table 1.** Studies on the impact of gut microbiota in the pathogenesis of IBD.

Research Issue	Type of research	Main findings in IBD	Research method	References	Significance
	Cross-sectional study	The richness and diversity of fecal microbiota ↓	16S rRNA Gene Sequencing	Alsulaiman^9^	
	Case-control study	Beneficial bacteria↓ Harmful bacteria↑	16S rRNA Gene Sequencing	Ahmadi^10^	
Microbial signature	Multicenter case control study	In CD patients, *Escherichia-Shigella*↑; In UC patients, *Enterococcus*↑	16S rRNA sequencing and metagenomics	Wang^11^	Dysbiosis of the microbiota may be associated with the onset of IBD.
	Cohort study	*Faecalibacterium*↑	16S rRNA Gene Sequencing	Gamboa^12^	
Metabolite signature	Case-control study	Energy metabolism-Ketone bodies↑	Proton nuclear magnetic resonance spectroscopy	García^13^	
	Case-control study	Amino acid metabolism-Differences and quantities of metabolites↑	Proton nuclear magnetic resonance spectroscopy	García^13^	Abnormal function of microbial metabolites may disrupt the intestinal barrier and promote inflammation.
		Fatty Acid metabolism- In active CD, palmitic acid↓, linoleic acid↑	Gas chromatography	Kikut^14^	
	Metabolomics research	Bile acid biosynthesis, vitamin digestion and absorption, and carbohydrate metabolism are affected	LC – MS/MS analysis;16S rDNA amplicon sequencing, Enrichment of metabolic pathways	Xu^15^	
Gut microbiome signature and stability Case-control study	Longitudinal study	With the passage of treatment time, the alpha diversit	Multi time point sampling,16S rRNA gene sequencing	Carlsen^16^	The decrease in microbial stability may drive IBD recurrence or progression
	Multi omics research	Microbial stability↓	Macrogenomics	Lee^17^	
	Case-control study	The stability of fungi changes in some IBD patients	Shotgun stool metagenomes	Guzzo^18^	
Transfer experiments in germ-free mice	Animal model research	Mice transplanted with CD patient microbiota exhibit stronger inflammatory responses	FMT	Sheikh^19^	Microbial communities have direct pathogenic or protective effects
	Animal model research	Reduction of colitis in sterile mice transplanted with healthy donor microbiota; Inflammatory markers↓	FMT,16S rRNA sequencing. LC-MS	Yin^20^	
	Animal model research	After transplanting IBD related microbiota, inflammation↑	Transfer experiment; transgenic mice	Niu^21^	
Single microbiome	Prospective case-control study	MAP was detected in the intestinal tissue of CD patients, and clinical trials of anti-MAP antibiotics for CD showed potential efficacy	Multivariable model	Kuenstner^22^	Specific pathogens may cause direct damage or participate in IBD
	Omics research	Map infection may participate in the onset of IBD by altering gut microbiota or triggering abnormal immune responses	Metagenomic analysis of the DNA	Elmagzoub^23^	
	Review	AIEC is closely related to the pathogenesis of IBD	–	Palmela^24^	
	Mechanism research	The colonization of AIEC causes changes in the gut microbiota and leads to the progression of inflammation	Animal model, Western blot analysis, Flow cytometric analysis, 16S rRNA gene sequencing, HE and PAS staining	Singh^25^	
IBD susceptibility genes	Research on the Interaction between Genetics and Microorganisms	More than 240 IBD related genetic loci, including the earliest discovered susceptibility gene NOD2, have been detected	Genome-wide association studies (GWAS)	Liu^26^，Hadad^27^，Cordero^28^，Liu^29^	Genetic susceptibility genes affect IBD risk by regulating host microbe interactions
	Animal model research	Immune related genes: IL-10,IL-15, IL-23 R, IL-12B, etc	Gene knockout model	Sakurai,^30^ Hurtubise^31^	
	Meta-analysis, review	Autophagy related genes: ATG16L1, IRGM, etc	–	Simovic,^32^ Larabi^33^	
	Mechanism research	Lysosomal regulatory genes: TFEB, etc	Gene knockout model	Zhang^34^	

The human gut microbiota fosters a symbiotic relationship with its host. In a state of health, the intestinal microbiota fulfills numerous vital physiological roles within the host, such as the digestion and metabolic processing of non-absorbable dietary components, the synthesis of essential vitamins, the modulation of the epithelial barrier, and the maturation of the intestinal immune system. The gut microbiome is pivotal in establishing and preserving the integrity of the intestinal mucosal barrier, as well as in sustaining the equilibrium of the intestinal microenvironment.^35^

### Characteristics of IBD-associated dysbiosis

Commensal bacteria play a vital role in regulating and maintaining the intestinal health of the host through diverse mechanisms. However, this balance can be disrupted, a condition referred to as “dysbiosis,” where the microbiota fails to provide its full range of beneficial effects, thus creating a favorable environment for the invasion of pathogenic bacteria. Research has demonstrated a correlation between the onset of IBD and intestinal dysbiosis.^36^ Understanding the composition of gut microbiota can help us predict the occurrence of diseases. In the prospective cohort study by Garay et al., it was found that gut microbiota can effectively predict the risk of CD onset, and high-risk populations can be accurately identified up to 5 y before the disease occurs.^37^ The dysbiosis observed in IBD patients is characterized primarily by a decline in beneficial bacteria, a proliferation of pathogens, and a decrease in microbial diversity. These gastrointestinal microbiota imbalances often share common microbiological features^38:^ (a). A reduction in bacteria that produce short-chain fatty acids (SCFAs); (b). A decrease in beneficial commensal bacteria with anti-inflammatory properties, which impairs the immune barrier of the intestinal mucosa; (c). An increase in the abundance of pathogenic bacteria, which induce inflammation via the secretion of endotoxins/pro-inflammatory factors.

### Etiological drivers of dysbiosis

Gut microbiota dysbiosis may be associated with various factors, including genetics, diet, and environmental exposures. These factors can trigger different degrees of gut microbiota dysbiosis, leading to the occurrence of enteritis. For example, research by A. Olivera et al. found that relatives from families with a high incidence of CD have a higher risk of developing CD, which may be due to the influence of environmental or genetic factors.^39^ And we all know that diet is the core regulatory factor for the composition and function of the intestinal microbiota. An imbalance in dietary fiber, fat, protein, and trace elements can easily lead to gut microbiota dysbiosis. Additionally, climate, dietary cultures, and pollutant exposure levels in different geographical regions may contribute to variations in microbiota characteristics. For instance, R Gacesa et al. found that the similarity of gut microbiota among family members decreases with prolonged separation, suggesting that geographical relocation may influence microbiota by altering environmental exposures (e.g., diet and pollutants).^40^ Overall, gut microbiota dysbiosis results from the interplay of multiple factors, including genetics, diet, environmental exposures, and geographical influences. Genetics may determine a host’s susceptibility to specific microbiota, while diet and environment directly modulate the composition of the microbiota.

Additionally, some studies have found that the oral microbiota may have a significant impact on the gut microbiota. For example, research has shown that periodontitis may induce gut microbiota dysbiosis through the salivary microbiota.^41^ This occurs as the oral microbiota enters the gut via swallowing (the gastrointestinal route) or through gingival damage caused by periodontitis, entering the bloodstream (the hematogenous route). Under normal circumstances, gastric acid and the intestinal barrier prevent the colonization of oral bacteria. However, in cases of gastric insufficiency, impaired intestinal barrier function, or after antibiotic use, the oral microbiota can easily colonize the gut. Furthermore, studies have discovered that certain bacteria from the oral cavity can not only directly colonize the gut but also lead to the accumulation of TH1 cells and inflammatory responses in the gut, which may be one of the mechanisms by which the oral microbiota contributes to IBD.^42^

### Decreased beneficial flora in the gut is the main pathogenesis of IBD

The primary characteristic of IBD dysbiosis is a reduction in overall species richness and alpha diversity. The depletion of beneficial bacteria and the loss of their protective functions are the most critical factors contributing to the development of IBD.^43^ Notably, the most significant changes include the decrease in the phylum *Firmicutes*, alongside a reduced abundance of the phylum *Bacteroidetes*.^44^ Within the *Firmicutes*, *Ruminococcus, Faecalibacterium prausnitzii*, and *Roseburia* are particularly relevant to the pathogenesis of IBD.^45,46^ These bacteria are the primary producers of intestinal SCFAs (especially butyrate). In a healthy state, they secrete butyrate to maintain the integrity of the intestinal barrier and regulate immune responses, thereby preserving intestinal homeostasis. Furthermore, studies have demonstrated that, compared to healthy individuals, the abundance of *Butyricicoccus, Lachnospiraceae, Akkermansia muciniphila, Blautia faecis, Roseburia inulinivorans, Coprococcus, Lactobacillus, Clostridium lavalense*, and *Bifidobacterium* is also diminished in the intestines of IBD patients ^47–50^ (Table 2).

**Table 2.** Overview of the gut microbiota in IBD patients (compared to healthy people).

IBD	Bacterium	Function in the gut	Abundance	Reference
UC	*Ruminococcus gnavus*	One of the most effective bacterial genera for carbohydrate decomposition, it stabilizes the intestinal barrier, reverses diarrhea, and increases energy. R. gnavus produces pro-inflammatory extracellular polysaccharides (EPS) that directly activate the host immune response. Its metabolites can influence the inflammatory response by regulating host gene expression.	Low	Crost^51^
UC & CD	*Faecalibacterium prausnitzii*	Acid and cholesterol transformations and fiber fermentation; Energy + anti-inflammatory metabolites production in the colon for the intestinal health. Prebiotic fermentation	Low	Lopez-Sile^52^
UC & CD	*Roseburia intestinalis*	Butyrate-producing bacteria. Prevents intestinal inflammation and maintains energy homeostasis by producing metabolites	Low	Pan^53^
UC & CD	*Butyricicoccus*	Adjust the balance of intestinal flora and promotes the proliferation of beneficial intestinal bacteria; it produces substances such as B vitamins and vitamin K, which have a health-promoting effect on the body.	Low	Olaisen^47^
UC & CD	*Lachnospiraceae*	Participate in the metabolism of various carbohydrates and have a strong ability to metabolize pectin (a complex dietary fiber and prebiotic) found in fruits and vegetables. Fermentation leads to the production of acetic acid and butyric acid, which are the main sources of energy for the host.	Low	Olaisen^47^
UC& CD	*Akkermansia*	The main secretor of mucin in the intestine, prevent invasion by harmful microbial flora.	Low	Zhang^54^
CD	*Lactobacillus reuteri*	Improve the distribution of intestinal flora and antagonizes the colonization of harmful bacteria.	Low	Petrella^55^
CD	*Coprococcus*	One of the main producers of butyrate, it suppresses immune responses and reduces the severity of allergic reactions.	Low	Yang^50^
CD	*Actinomyces*	Commensal inhabitants of the oral cavity and intestinal tract. Acquire pathogenicity through the invasion of breached or necrotic tissue. As the infection progresses, granulomatous tissue, extensive reactive fibrosis and necrosis, abscesses, draining sinuses, and fistulas are formed	High	Morgan^56^
CD	*Veillonella*	Resident flora of the oral cavity and intestine. They acquire pathogenicity by invading damaged or necrotic tissues.	High	Morgan^56^
UC& CD	*Intestinibacter*	Ferment glucose to produce acid or gas, catalase positive, oxidase negative, and can reduce nitrate to nitrite	High	Baldelli^57^
UC& CD	*E. coli*	Harmless strains are normal resident bacteria in the animal’s intestinal tract that produce vitamin K, prevent the growth of other pathogenic bacteria in the intestinal tract, and are beneficial to humans. Certain strains can utilize their flagellar antigens and release toxins to cause disease.	High	Morgan^56^

#### Immunomodulatory effects of commensal microbes

The aberrant immune response of the host serves as the principal pathogenic mechanism underlying the development of IBD. The gut microbiota can mitigate the progression of IBD by modulating various immune responses. This primarily occurs via:

(a). Aberrant activation of the nuclear factor-κB (NF-κB) signaling pathway has been shown to be an important feature of the inflammatory response in IBD. Clinical data have shown that constitutive activation of NF-κB in the colonic tissues of IBD patients leads to sustained overproduction of pro-inflammatory factors (e.g., TNF-α and IL-1β), creating a vicious inflammatory cycle.^58^ Notably, specific probiotic strains demonstrated the ability to modulate this pathway: by disrupting the endoplasmic reticulum stress response, *L. acidophilus* effectively inhibited the aberrant activation of NF-κB, and a significant reduction in the level of the pro-inflammatory factor TNF-α was observed in the DSS-induced mouse model of colitis, while the secretion of the anti-inflammatory factor IL-10 secretion increased 2.3-fold.^59^ This bidirectional regulatory effect was further validated in studies with *L. rhamnosus*, which significantly improved intestinal barrier integrity by inhibiting the TLR4/NF-κB signaling cascade and reducing the expression of the key chemokine CXCL1–5.^60^

Specific strains can exert anti-inflammatory effects by regulating the Janus kinase/signal transducer and activator of transcription (JAK/STAT) signaling pathway: experimental data show that some probiotics can inhibit the activation of the phosphorylation of STAT1 and STAT3, which can effectively alleviate the intestinal inflammatory response.^61^ Notably, different bacterial strains have specificity in regulating the STAT signaling pathway, for example, *Lactobacillus* were found to significantly promote IL-10 secretion from mesenchymal stem cells (MSCs) through activation of the STAT3 signaling pathway, a mechanism that not only enhances immune regulation but also effectively improves the pathologic phenotype of mice in a model of colitis.^62^ In addition, intervention experiments with *L. casei* showed that the strain ameliorated DSS-induced colitis in mice through a dual mechanism of action-both by inhibiting the pro-inflammatory JNK/p-38 signaling pathway and by regulating gene expression through the enhancement of the acetylation level of the histone H3K9 locus.^63^

(b). At the level of immune cell regulation, intestinal *Lactobacillus* was found to promote the maturation and differentiation of dendritic cells and enhance the activity of natural killer (NK) cells, in which interferon-gamma (IFN-γ) produced by NK cells plays a key role in the initiation of Th1-type immune responses in lymph nodes. Notably, clinical observations showed that *Lactobacillus abundance* was significantly reduced in the intestines of CD patients, while animal experiments confirmed that specific Lactobacillus strains effectively inhibited the over-synthesis of IL-6 and IFN-γ in chronic IBD model mice.^64^

In terms of cytokine regulation, specific flora such as *Prevotella* and *Akkermansia* were significantly correlated with elevated intestinal IL-10 levels, and the mechanism may be related to the promotion of the differentiation of regulatory T-cells (Tregs), which are the main secretory cells for the anti-inflammatory factor IL-10.^65,66^

(c). Some probiotic strains also exhibit direct immunomodulatory properties. For example, *Lactobacillus* and *Bifidobacterium* can modulate immune responses in the local microenvironment of the gut by releasing cell wall components (e.g., peptidoglycan) and microbe-associated molecular patterns (MAMPs) such as bacterial DNAt.^67^ These flora not only enhance phagocytic activity and NK cell function but also maintain immune homeostasis by inducing apoptosis of activated T cells.^68^

**Figure 1.**
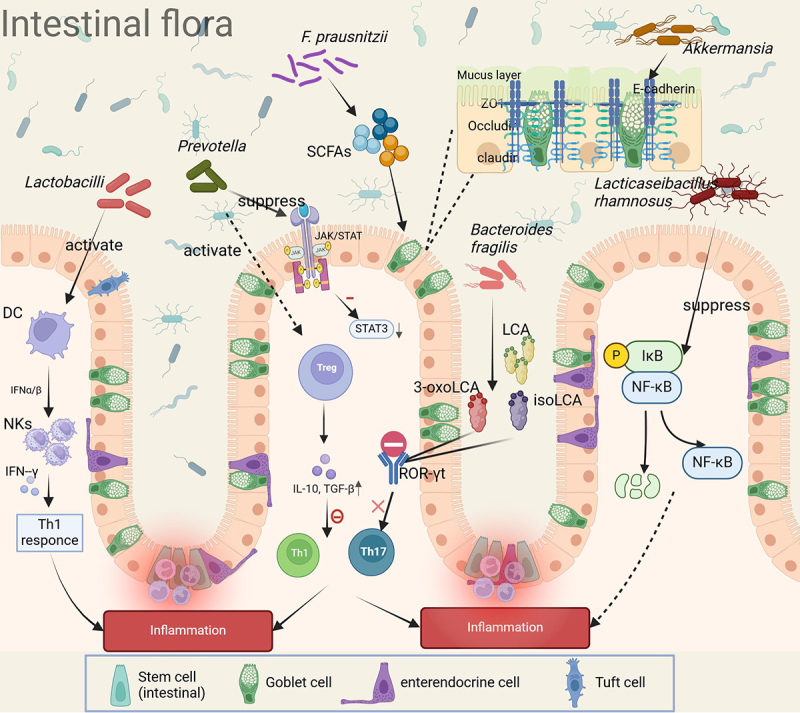
Gut flora mitigates IBD in a variety of ways (modulation of immune response, mucosal barrier, and regulatory effects of metabolites).

In terms of the overall regulatory mechanism, the gut microbiota maintains cytokine network homeostasis by regulating the balance between Tregs and Th1/Th17 cells and typical representatives, such as *F. prausnitzii*, can play a protective role in the intestinal tract by inducing the differentiation of Tregs, which can significantly elevate the level of anti-inflammatory factors, such as IL-10.^69^ The above findings suggest that restoring the normal response pattern of the host immune system by targeting the intestinal flora composition and its metabolic function may become an important strategy for the prevention and treatment of IBD (Figure 1).

#### Barrier function restoration by probiotics

One of the core pathological features of IBD is intestinal mucosal barrier dysfunction. Studies have shown that probiotics such as *F. prausnitzii* and *L. rhamnosus* can improve intestinal barrier integrity through multiple pathways. Their mechanisms of action include regulating paracellular permeability, up-regulating the expression levels of occludin and E-cadherin, and maintaining the structural stability of the barrier by stimulating the gut microbiota to produce mucins and host antimicrobial peptides.^70,71^ Notably, probiotics are particularly effective in promoting the expression of tight junction proteins (TJs), for example, *L. rhamnosus* and *L. plantarum* can enhance intestinal barrier function by up-regulating the expression of ZO-1 and occludin.^72,73^ Further studies revealed that *L. rhamnosus n*ot only prevented cuprocyte loss but also specifically promoted the expression of ZO-1, occludin and claudin-13.^62^ The significant depletion of *A*. mucinophilic, a key mucus-secreting bacterium of the gut, is of concern in the pathologic course of IBD. Zhang et al.^54^ found that the abundance of this bacterium in the intestinal tract of IBD patients was significantly lower than that of healthy people through comparative analysis, and this bacterial imbalance would lead to the destruction of the structure of the collagen layer of the intestinal mucosa, which would exacerbate the invasion of the intestinal epithelium by harmful fecal toxicants.

#### Metabolites

Another major negative effect of the absence of beneficial intestinal bacteria is a decrease in anti-inflammatory metabolites. Metabolites of intestinal flora, especially butyrate and bile acid derivatives, may exert anti-inflammatory effects through interactions with immune cells. *Butyrate* is produced by commensal bacteria such as *B. thetaiotaomicron* and *F. prausnitzii* and exhibits significant anti-inflammatory properties in IBD. Its mechanism of action includes inhibition of HDAC activity, induction of differentiation of Tregs and promotion of anti-inflammatory cytokine secretion. In addition, butyrate enhances the antigen tolerance of intestinal epithelial cells, reduces the level of pro-inflammatory factors, and maintains intestinal homeostasis by regulating the composition of the bacterial flora and reinforcing the mucus layer.^74–77^

Bile acid metabolism, another important immunomodulatory pathway, and its derivatives regulate gene expression related to TH differentiation.^78^ Primary bile acids (PBA) synthesized by the liver are biotransformed by the intestinal flora to produce secondary bile acids (SBA), a metabolic process that is closely related to structural changes in the bacterial flora and has a direct impact on the composition of the bile acid pool and disease regression.^79,80^ A key feature of IBD is the significant reduction in SBA content. In UC and CD, the host-produced PBA are elevated relative to the microbiota-modified SBA and their downstream metabolites.^81^ *P. fragilis* catalyzes the conversion of LCA to isoLCA and 3-oxoLCA via 3α-hydroxysteroid dehydrogenase, two metabolites that exert anti-inflammatory effects by blocking the retinoic acid receptor-associated orphan receptor γt (ROR-γt) pathway, inhibiting Th17 cell differentiation and decreasing levels of the pro-inflammatory factor IL-17.^78^ The regulatory effects of these metabolites provide potential directions for developing new treatment strategies, particularly for diseases related to immune dysregulation.

### Increase of gut pathogenic bacteria in IBD

The diminution of advantageous gut microbiota in IBD patients results in a compromised immune barrier function, facilitating the intrusion of detrimental pathogens and intensifying inflammatory infiltration. This is predominantly manifested by an augmentation in the prevalence of *Proteobacteria*.^56^ A plethora of studies have demonstrated that *Enterobacteriaceae*, a member of the *Proteobacteria* phylum, are the principal pathogenic bacteria inducing IBD.^57,82^ Furthermore, an elevation in *Enterobacteriaceae* is linked to an increased risk of therapeutic failure in CD patients,^83^ which might be associated with their influence on intestinal mucosal healing and repair.

A typical example within *Enterobacteriaceae* is the epithelium-associated invasive *Escherichia coli*, known as *adherent-invasive Escherichia coli* (*AIEC*). This section is detailed in “Infectious Colitis”. Furthermore, there are opportunistic pathogens such as *enterotoxigenic Bacteroides fragilis* (*ETBF*), which promote inflammation in IBD patients by producing inflammatory mediators like adhesins and *Bacteroides fragilis toxin* (*BFT*).^84^ The main toxin of *ETBF* and *BFT*, is a metalloprotease that can destroy the integrity of intestinal epithelial cells, triggering an inflammatory response.^85^ Studies have shown that *BFT* induces the activation of a series of pro-inflammatory signaling pathways, including IL-17 R, NF-κB, and STAT3, through its interaction with intestinal epithelial cells.^86^ The pathogenicity of *ETBF* extends beyond the direct effects of its toxin and includes complex interactions with the host immune system. Research has found that ETBF can exacerbate inflammatory responses by modulating the host’s immune response, particularly by influencing the production of cytokines (mainly IL-6, IL-8, IL-1β, and TNF-α).^87^

### The impact of fungi on IBD

While the global consensus recognizes the role of bacterial dysbiosis in inducing IBD, recent years have seen increasing attention on the contribution of intestinal fungi to IBD pathogenesis.^88^ Although bacteria dominate the gut microbiota, fungi, despite their lower abundance accounting for only 0.01%–0.1% of the gut microbiome, play a decisive role in maintaining intestinal flora balance and mucosal immune responses.^89,90^ At the phylum level, *Ascomycota* and *Basidiomycota* are the most common in the gut. At the genus level, the fungal community is mainly composed of *Saccharomyces*, *Aspergillus*, *Candida*, *Debaryomyces*, *Malassezia*, *Penicillium*, and *Pichia*, among which *Saccharomyces* is the most abundant.^91^

Disease-associated alterations in fungal composition exhibit distinct patterns across IBD subtypes. Active disease states correlate with elevated *Basidiomycota/Ascomycota* ratios compared to remission phases and healthy controls. Species-level analysis reveals significant mycobiota shifts characterized by increased *Candida* species (including *C. albicans*, *C. tropicalis*, *C. lusitaniae*, *Cyberlindnera jadinii*, and *Kluyveromyces marxianus*) alongside diminished *Saccharomyces* populations in IBD patients.^92^ Particularly in UC, *C. albicans* demonstrates pathogenic potential through mucosal adherence and Th17-mediated inflammatory responses. Corticosteroid therapy may exacerbate this process by compromising colonization resistance, enabling fungal proliferation within the IBD inflammatory microenvironment.^93^

CD exhibits unique fungal signatures, with elevated levels of *C. tropicalis* demonstrating synergistic biofilm formation with enteric bacteria (*E. coli* and *Serratia marcescens*), potentially amplifying intestinal inflammation.^94^ *Malassezia restricta* is also a fungus that is more abundant in patients with CD. It primarily triggers a strong inflammatory response in patients through a signal adaptor protein, CARD9, which is crucial for antifungal defense. Additionally, *Malassezia restricta* can stimulate immune cells to produce more inflammatory factors, such as TNF-α and IL-8, thereby exacerbating intestinal inflammation.^95^ *Debaryomyces hansenii* is another fungus commonly found in CD patients, which inhibits the proliferation of fibroblasts and macrophages, leading to impaired healing of intestinal wounds. In addition, this fungus can further exacerbate the intestinal inflammatory response by inducing the expression of the cytokine CCL5.^96^ These fungal compositional changes collectively suggest mycobiota dysregulation as a potential pathophysiological mechanism in IBD. Mechanistic studies reveal multiple fungal pathogenic pathways, including intestinal barrier disruption via epithelial junction protein impairment, enhanced drug resistance through biofilm formation, and immunomodulatory effects.^97^ Such mechanisms may synergize with bacterial dysbiosis to perpetuate intestinal inflammation.

Current therapeutic strategies targeting gut microbiota modulation show promise, particularly probiotics, prebiotics, and fecal microbiota transplantation. While preliminary results are encouraging, rigorous evaluation of long-term safety and therapeutic efficacy remains essential for clinical translation. Future research directions should focus on elucidating fungus-bacteria-host interactions and developing mycobiota-specific interventions to complement existing IBD management approaches.

### Gut virome in IBD

The gut virome, a crucial component of the gut microbiota, primarily consists of bacteriophages and eukaryotic viruses. Recent studies have revealed a significant imbalance in the gut virome of patients with IBD,^98^ characterized by an increased abundance of *Caudovirales* and a reduction in *Microviridae*. This imbalance is associated with decreased gut microbiota diversity and enhanced inflammatory responses. *Orthohepadnavirus genus viruses*, such as the *hepatitis B virus*-associated protein (*HBx)*, may contribute to the pathogenesis of UC by interfering with intestinal epithelial cell function and activating immune responses. Viral dysbiosis may influence the progression of IBD through the following mechanisms: (a) Disruption of the intestinal barrier: Phage lysis of host bacteria releases bacterial components (such as LPS), which activate host immune receptors (such as TLR4), exacerbating inflammation. (b) Direct regulation of immune cells: Virus-like particles (VLPs) can induce the secretion of pro-inflammatory factors, aggravating colitis symptoms. Additionally, the virome is closely associated with the dynamic equilibrium of bacterial communities. For instance, bacteriophages regulate microbial composition by lysing specific bacteria (e.g., *Enterobacteriaceae*), while abnormal proliferation of bacteriophages in IBD may lead to a reduction in beneficial bacteria (e.g., *butyrate-producing Ruminococcaceae*).^99^ Viral genes may also participate in host metabolic pathways (e.g., sulfur metabolism and oxidative stress), further influencing intestinal homeostasis.^100^

The impact of the gut virome on intestinal immunity and barrier function has garnered increasing research attention, yet inter-individual and methodological variations limit the generalizability of IBD-associated virome biomarkers. Although phage therapy is currently primarily confined to compassionate use, it has demonstrated promising therapeutic potential in animal models and preclinical studies. These findings provide new references for the diagnosis and treatment of intestinal diseases, advancing a deeper understanding of the role of the gut virome in health and disease.

### Necrotizing enterocolitis (NEC)

NEC is the most severe gastrointestinal emergency in preterm infants, with persistently high morbidity and mortality rates, posing a significant challenge in the field of neonatal intensive care. Although its pathophysiological mechanisms have not been fully elucidated since its first report in the nineteenth century,^101^ recent studies have shown that dysbiosis of the gut microbiota plays a central role in the development of NEC. Early changes in microbial communities may affect the integrity of the intestinal barrier and activate a series of cytokines, leading to excessive inflammatory responses in the intestinal wall tissue, which can result in localized or diffuse necrosis of intestinal epithelial cells.^102,103^

#### The epidemiological association between NEC and intestinal dysbiosis

The composition and diversity of the gut microbiota in preterm infants significantly differ from those in healthy full-term infants, and this disparity provides a “susceptible soil” for the development of NEC. Microbiome analysis reveals that the gut microbiota of NEC patients exhibits characteristic alterations: an increased relative abundance of *Proteobacteria*, and a decreased abundance of protective *Firmicutes* and *Bacteroidetes* .^104^ Among these, the abnormal proliferation of *Clostridium spp*. is positively correlated with the severity of NEC, while the depletion of *Bifidobacterium* and *Lactobacillus* weakens the intestinal immune defense capabilities.^105,106^ It is noteworthy that this dysbiosis is not dominated by a single pathogen but is the result of synergistic actions of multiple bacterial species.^107^ Shaw’s research introduced a microbial community pattern: the gut microbiota triggers NEC due to imbalanced stimulation of TLR4 and TLR9. For instance, the reduction of CpG motifs in the genomic DNA of *C. perfringens* may attenuate the anti-inflammatory protection mediated by TLR9, promoting TLR4 signaling and leading to epithelial necrosis, while *Cronobacter sakazakii (C. sakazakii)* exacerbates intestinal epithelial damage by activating the TLR4/MyD88/NF-κB signaling axis.^108,109^

### Molecular mechanisms of intestinal barrier disruption by dysbiosis

The integrity of the intestinal barrier relies on the stable expression of TJs (such as ZO-1 and occludin), whose function is directly regulated by the composition of the gut microbiota.^110^ When microbial diversity decreases, intestinal permeability can increase several-fold, leading to the translocation of pathogens and endotoxins, which triggers excessive inflammatory responses, a process particularly prominent in the pathological progression of NEC. Taking *C. sakazakii* as an example, this Gram-negative pathogenic bacterium disrupts the intestinal epithelial barrier by inhibiting the expression of ZO-1 and occludin, significantly increasing the risk of NEC.^111^

However, specific probiotics can reverse barrier damage through multiple mechanisms. *Ligilactobacillus salivarius* derived from breast milk can restore the mRNA and protein levels of tight junction proteins in *C. sakazakii*-infected cells, while a similar probiotic mixture can also activate the PXR-JNK signaling axis, upregulate GADD45β expression, thereby inhibiting JNK phosphorylation and alleviating LPS-induced barrier disruption.^112,113^ Similarly, *Clostridium tyrobutyricum* significantly improves intestinal integrity in NEC model animals by upregulating the expression of mucosal barrier-related genes such as ZO-1 and Muc2, whereas *C. butyricum* exacerbates barrier dysfunction due to its competitive inhibition of *A. muciniphila* colonization.^114^ *Bifidobacterium infantis* reduces intestinal permeability in NEC mice by regulating the internalization of claudin-4 and occludin^115^; Maternal supplementation with this strain also enhances ZO-1 expression in offspring, promoting the formation of tight junctions in pre-weaning pups.^116^ Notably, strain-specific effects are significant: although both belong to *Clostridium*, *C. tyrobutyricum* and *C. butyricum* have opposite impacts on barrier function. These findings collectively indicate that the microbial-barrier dynamic equilibrium is a key target for NEC prevention and treatment.

### Inflammatory regulatory networks in colony-immune interactions

The gut microbiota maintains continuous communication with the host immune system through pattern recognition receptors (PRRs), a process that appears to be dysregulated in NEC, with the overactivation of TLR4 seemingly acting as the core driver of the inflammatory storm in NEC. Negative regulation of the TLR4 pathway has emerged as a critical target for NEC intervention. *L. rhamnosus* alleviates TLR4-mediated intestinal injury through dose-dependent upregulation of TLR inhibitors SIGIRR and A20^117^; *B. adolescentis* significantly reduces NEC in preterm rats induced by hypoxia and cold stress by increasing TOLLIP and SIGIRR expression.^118^ Additionally, *S. boulardii* mitigates intestinal injury in neonatal NEC mice by inhibiting the SirT1/NF-κB axis, suggesting the cross-species regulatory potential of fungal probiotics.^119^

It is noteworthy that strain-specific effects significantly influence the intervention outcomes, similar to the impact on intestinal barrier function. *C. tyrobutyricum* inhibits inflammation by downregulating the TLR4/NF-κB pathway, whereas its counterpart, *C. butyricum*, possesses the capability to exacerbate inflammation.^114^ Maternal intervention studies have further expanded the therapeutic window parental supplementation with *L. acidophilus* and *B. infantis* can upregulate the expression of vitamin D receptor (VDR) in offspring, inhibit p65 phosphorylation, and reduce NF-κB signaling activity, thereby alleviating intestinal inflammation.^120^

### Inflammatory regulatory networks in colony-immune interactions

The metabolites of gut microbiota play a “double-edged sword” role in NEC, with bile acid metabolism disorder being the central link: Taurochenodeoxycholic acid (TCDCA) significantly exacerbates intestinal epithelial damage by activating the farnesoid X receptor (FXR) and the NLRP3 inflammasome; whereas *B. fragilis* converts TCDCA into a nontoxic form through bile salt hydrolase (BSH) activity, reducing FXR signaling activity, thereby inhibiting the inflammatory pathway and alleviating the pathological progression of NEC.^121^ However, certain strains such as *C. scindens* in the *Clostridium* genus increase the severity of NEC in neonatal rats by upregulating the ileal bile acid transporter Asbt, promoting the accumulation of toxic secondary bile acids.^122^

In addition to bile acids, SCFAs also exhibit antagonistic effects against toxic metabolites. The intestinal butyrate level decreases in NEC patients, and butyrate supplementation can inhibit immune responses by inducing Treg differentiation, thereby alleviating intestinal inflammation and tissue damage.^123^ In contrast, the formate produced by *Enterobacter cloacae* and *Klebsiella pneumoniae* exhibits dual toxicity: on the one hand, it inhibits cellular respiration, leading to energy depletion in intestinal epithelial cells; on the other hand, it promotes cytotoxicity, inducing necroptosis and resulting in enteritis. Moreover, multi-omics analysis reveals a significant positive correlation between fecal formate levels and the severity of intestinal injury in NEC patients.^124^

Intestinal microbiota dysbiosis plays a pivotal role in the pathogenesis of NEC by disrupting barrier function, activating inflammatory pathways, and altering metabolic profiles. Although existing evidence supports the clinical potential of probiotics and FMT, their efficacy is constrained by multiple factors such as strain specificity, individual variations, and intervention timing. Future directions include (a) establishing NEC risk prediction models based on metagenomic/metabolomic features; (b) developing targeted inhibitors against toxic metabolites (e.g., formate); (c) exploring intervention strategies for FMT. Through the interdisciplinary integration of microbiome science, immunology, and synthetic biology technologies, a new era in the prevention and treatment of NEC is anticipated.

## Radiation colitis (RC)

The widespread use of ionizing radiation (IR) in cancer radiotherapy has led to radiation enteritis (RE) as the most common complication in patients with abdominopelvic tumors.^125^ Despite innovations in radiotherapy techniques, rapidly proliferating intestinal epithelial cells remain susceptible to radiation toxicity, triggering noninfectious intestinal inflammation by mechanisms involving complex interactions of multiple cell types (e.g., endothelial cells) and molecular signals (e.g., cytokines).^126^ The pathological process can be divided into five phases: the ROS-mediated DNA damage phase, the inflammatory/apoptotic phase, the signal amplification phase, the ulcer formation phase and the healing phase.^127^ In recent years, radiation-induced dysbiosis (dysbiosis) of the intestinal flora has been gradually recognized as a key driver of RC development through 16S rRNA sequencing, and the influence of environmental factors on the flora is significantly stronger than that of the host genetic background.^128,129^

### Radiation-induced microbial imbalances: from phenomena to mechanisms

**Figure 2.**
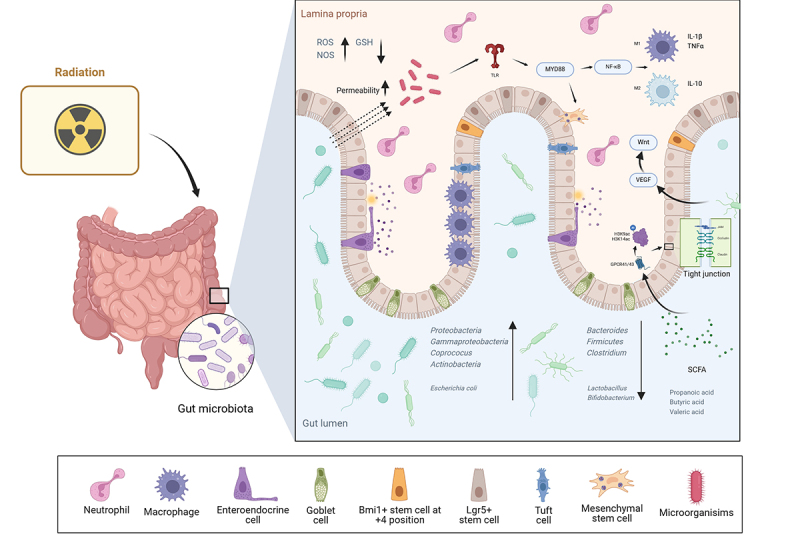
Radiation causes colitis through gut flora dysbiosis.

Perturbation of the intestinal flora by radiotherapy is dose-dependent and site-specific. Clinical cohort studies have shown that among gynecologic oncology patients receiving pelvic radiotherapy, those who developed severe diarrhea retained only 29% similarity in gut microbial composition compared to healthy controls, compared to 60% of those who did not have diarrhea.^130^ The same evidence was found in a study by Wang et al. The diversity of microbiota composition was reduced in both the diarrhea and non-diarrhea groups as a result of radiation therapy, and the reduction in diversity was greater in the diarrhea group.^131^ This imbalance showed a characteristic pattern: an increase in the abundance of conditionally pathogenic bacteria (e.g., *E. coli*) of *Proteobacteria* and *Actinobacteri*, and a decrease in commensal bacteria (e.g., *Lactobacillus*) of *Firmicutes* and *Bacteroidetes*.^125^ This imbalance results in conditioned pathogens that will colonize and take over the ecological niche, promoting increased endotoxin release, disrupting the intestinal epithelial barrier and exacerbating inflammation (Figure 2). Notably, disturbed gut flora (as *Coprococcus* enriched) prior to radiotherapy may enhance RC susceptibility.^132^

### Microbial imbalance: a key intermediate in the pathogenesis of RC

Abdominal radiotherapy was also found to disrupt gut flora homeostasis and significantly reduce gut microbiota diversity in mice in an animal model.^133^ Notably, dysbiosis is not a passive consequence of radiation, but actively participates in the RC process: FMT experiments demonstrated that early correction of the flora structure ameliorated symptoms of colitis such as diarrhea and pain, as well as mucosal damage, and enhanced radiation resistance by modulating host mRNA/lncRNA expression.^134,135^

The absence of key commensal bacteria is particularly prominent in RC. *A. muciniphila* is the most severely reduced genus after radiation and exerts its protective effects through two complementary mechanisms: (a) secretion of propionic acid activates the GPR43 receptor, which promotes continuous renewal of the mucus layer and expression of tight junction proteins, preserving the integrity of the intestinal epithelial barrier^135^; (b) driving M2 macrophage polarization and improve inflammatory factor levels.^136^ This suggests that dual metabolic-immune regulation of specific strains may be an important target for RC therapy.

Although flora regulation provides new ideas for RC treatment, there are still key issues that need to be addressed: first, baseline flora disorders caused by cancer itself may affect the effectiveness of the intervention, and individualized therapeutic strategies based on macro-genomic signatures need to be established; second, microenvironmental changes in the radiation gut (e.g., thinning of the mucus layer and elevation of the pH) may limit the colonization of probiotics. Future studies need to integrate multi-omics technologies to elucidate the metabolite-epigenetic regulatory network of the flora and promote RC treatment in the era of precision medicine. Intestinal flora plays the dual role of “amplifier” and “regulator” in RC. From the analysis of pathogenic mechanisms to the development of intervention strategies, microbiome research is profoundly changing our understanding of radiation bowel injury. Through targeted regulation of bacterial homeostasis, it is expected that the biological barrier of intestinal protection can be built for patients while guaranteeing the anti-cancer effect of radiotherapy.

### Drug-induced colitis (DC)

DC is an inflammatory disease of the colon triggered by specific medications, with pathological features identifiable through endoscopic or histological examination. Although relatively rare in clinical practice, the importance of etiological diagnosis for this condition has become increasingly prominent with the development and widespread use of novel drugs. Currently, epidemiological data on DC remain elusive; however, discontinuation of the offending agent often induces clinical remission.^137^ Recent studies have untangled a critical role for gut microbiota dysbiosis in DC. Medications not only directly damage the intestinal epithelium but also indirectly exacerbate inflammation by disrupting microbial metabolic networks (Table 3).

**Table 3.** Studies on drug-induced colitis.

Drug	Description of the drug	Type of research	Date of publication	Author
Leflunomide	Antirheumatic drug	Case report	2024	Ebrahim^138^
Mycophenolate mofetil	Immunosuppressive medication	Case report	2024	Berake^139^
Eplerenone	Aldosterone receptor blocker	Case report	2023	Saito^140^
Secukinumab	IL-17 monoclonal antibody inhibitor	Case report	2022	Gandu^141^
Encorafenib and binimetinib	BRAF and MEK inhibitors	Case report	2022	Gelsomino^142^
Osimertinib	Third-generation epidermal growth factor receptor tyrosine-kinase inhibitor	Case report	2022	Wang^143^
Ipilimumab and nivolumab	Immune checkpoint inhibitors	Case report	2021	Moein^144^
Levodopa	Parkinson’s disease drug	Case report	2018	Zanelli^145^
Idelalisib	Selective inhibitor of the delta isoform of phosphatidylinositol 3-kinase	Case report	2017	Hammami^146^
Pembrolizumab	Anti-PD1	Case report	2016	Baroudjian^147^
Olanzapine	Atypical antipsychotic drugs	Case report	2016	Fernandes^148^
Trientine	Copper-chelating agent	Case report	2015	Boga^149^
Phenytoin and lamotrigine	Antiepileptic	Case report	2014	Swanson^150^
Amoxicillin-clavulanate	Antibiotic	Case report	2010	Hoffmann^151^
5-fluorouracil	Anti-cancer drugs	Case report	2004	Akay^152^
NSAIDs		RCT	2009	Geramizadeh^153^
Tacrolimus	Immunosuppressant used in solid organ transplant recipients.	Retrospective study	2022	Hissong^154^
Anti-PD-(L)1 and anti-CTLA4	Immune checkpoint blockade	Retrospective study	2021	Luo^155^
NSAIDs		Retrospective study	2010, 2009	Deshpande^156^ Casella^157^
5-fluorouracil	Anti-cancer drugs	Retrospective study	2002	Madisch^158^
NSAIDs		Retrospective study	2000	Püspök^159^
Neuroleptic	Antipsychotic drug	Prospective study	2016	Abdalla^160^

#### Antibiotics

Antibiotic exposure significantly increases the risk of colitis by disrupting the homeostasis of the gut microbiota. The core mechanism involves a decrease in colonization resistance due to the depletion of commensal bacteria.^161,162^ Clinically, this manifests as diarrhea (with an incidence rate of up to 25%), typically appearing 4–9 d after medication. This condition is closely associated with a reduction in SCFAs, abnormal fermentation of carbohydrates, and overgrowth of opportunistic pathogens (such as *Candida* and *Gram-negative bacteria*) triggered by microbiota dysbiosis.^163^ It is worth noting that maternal antibiotic use can induce gut microbiota dysfunction in offspring through vertical transmission, reducing microbial diversity and enhancing susceptibility to colitis. This phenotype can be reversed by early supplementation with key commensal bacteria.^164,165^

Antibiotic-induced microbiota imbalance significantly weakens the intestinal ecological barrier, allowing drug-resistant pathogens like *Salmonella enterica* and *Clostridium difficile* to gain a colonization advantage.^166^ Among them, CDI is a major cause of antibiotic-associated colitis. Although *C. difficile* itself is not life-threatening, human intervention can turn it into a significant contributor to colitis morbidity and mortality. Its high drug resistance (such as resistance to third-generation cephalosporins and carbapenems) and toxin synergism can lead to severe complications such as toxic megacolon.^167,168^ Additionally, a special form of antibiotic-induced drug colitis is the abnormal proliferation of cytotoxin-producing *Klebsiella oxytoca* induced by penicillin treatment, which triggers characteristic hemorrhagic colitis (AAHC). This condition is characterized by the absence of *C. difficile*, and its pathophysiology may be related to allergic reactions, mucosal ischemia, and direct damage by bacterial toxins.^169,170^

Metabolic competition within the gut microbiota serves as a crucial mechanism for defending against pathogens: Commensal bacteria restrict the proliferation of pathogenic bacteria by occupying nutritional niches.^171^ Antibiotics disrupt this microecological balance, allowing pathogens to acquire sufficient metabolic substrates and breach colonization thresholds, ultimately triggering colitis. This process untangles the essence of drug-induced colitis a cascade of pathological reactions initiated by the collapse of microbial homeostasis.

#### Non-steroidal anti-inflammatory drugs (NSAIDs)

NSAIDs are the second major cause of drug-induced colitis after antibiotics,^172^ and their mechanisms of colonic injury involve multi-dimensional pathological changes. Clinical observations have shown that NSAIDs, such as aspirin and indomethacin, can trigger a broad spectrum of colonic pathologies, including ulcers, eosinophilic colitis, ischemic colitis, and microscopic colitis.^153,163^ The widespread use of sustained-release preparations has further exacerbated the incidence of NSAIDs-induced enteropathy.^173^

Existing research clearly suggests that intestinal flora imbalance is the core mechanism of NSAIDs-induced intestinal inflammation.^174^ Long-term NSAIDs users exhibit multi-dimensional pathological features of intestinal damage, ranging from mucosal erosion and increased permeability to ulcer formation, as well as more severe clinical outcomes.^175^ Microbiome analysis has revealed that NSAIDs exposure significantly alters the structure of the flora: Patients experience abnormal fluctuations in the abundance of *Prevotella*, *Bacteroides*, *Ruminococcaceae*, and *Barnesiella*, while ibuprofen specifically induces the enrichment of *Propionibacteriaceae*, *Pseudomonadaceae*, and other flora.^176^ In the elderly population, NSAIDs can lead to a decrease in the abundance of *Collinsella*, *Actinobacteria*, and *Lactobacillus*, exacerbating intestinal epithelial repair disorders.^177^ Gender differences are particularly significant in NSAIDs-induced enteropathy: in an indomethacin-induced acute injury model, the diversity of intestinal flora in female patients decreases compared to males, accompanied by an increase in *Actinobacteria* abundance and an imbalance in the proportion of *Bacteroidetes* and *Proteobacteria*.^178^ Animal experiments further confirm that indomethacin treatment can promote the amplification of *Gram-negative bacteria* (such as *Bacteroides, Enterobacteriaceae*, and *Clostridium*), aggravating intestinal inflammation through endotoxin release.^179^ Intervention studies have shown that daily supplementation with *Bifidobacterium* can reduce the incidence of NSAIDs-related intestinal ulcers in healthy volunteers, suggesting the potential therapeutic value of targeted flora regulation.^180^

The abnormal proliferation of *Gram-negative bacteria* and *Anaerobic bacteria* is a key driving factor in the progression of NSAIDs-induced enteropathy. These flora increase intestinal permeability by releasing endotoxins and organic acids, promoting bacterial translocation and aggravating mucosal damage.^174^ Clinical epidemiological data confirm that NSAIDs use increases the risk of CDI, and its mechanism involves flora imbalance and toxin synergy.^181,182^ Additionally, *Adherent-invasive Escherichia coli* (*AIEC*) activates NLRP3 inflammasome and Caspase-8 in an NSAIDs-exposed IL10^−/−^ mouse model, inducing pyroptosis and apoptosis of epithelial cells, leading to a collapse of barrier function.^25^

The TLR4-MyD88-NF-κB signaling axis plays a central regulatory role in NSAIDs-induced enteropathy. LPS derived from *Gram-negative bacteria* is recognized by TLR4, activating the MyD88-dependent pathway, promoting NF-κB nuclear translocation and releasing cytokines, driving neutrophil infiltration.^183,184^ Gene knockout studies have confirmed that TLR4^−/−^ and MyD88^−/−^ mice are completely resistant to indomethacin-induced intestinal damage and can significantly inhibit cytokine expression.^185^

Disorders of intestinal flora metabolites further exacerbate the pathological process. Long-term NSAID use reduces the concentration of SCFAs (such as isobutyric acid and isovaleric acid) in the feces of elderly individuals, leading to intestinal epithelial energy metabolism disorders.^186^ SCFAs activate the NLRP3 inflammasome and promote IL-18 secretion through the GPR43 receptor, thereby improving intestinal barrier integrity.^187^ It is worth noting that the tryptophan metabolite, indole, can improve NSAID-induced enteropathy by regulating the innate immune response: in indomethacin-treated mice, exogenous indole intervention reduces *Bacteroidales* abundance while promoting *Clostridiales* proliferation.^188^ These findings untangle the pivotal role of the flora-metabolism-immune interaction network in NSAIDs-induced enteropathy.

#### Antitumor drugs

With the rapid development of antitumor therapies, the incidence of drug-induced colitis has significantly increased, severely affecting patients’ quality of life and leading to treatment interruptions. This is particularly evident with immune checkpoint inhibitors and traditional chemotherapeutic drugs. These drugs fight tumors by modulating the immune system but may also trigger autoimmune reactions, resulting in the occurrence of colitis.^189,190^

Irinotecan (CPT-11), a topoisomerase I inhibitor, promotes macrophage infiltration into intestinal tissues and activates the NLRP3 inflammasome, facilitating the production of IL-1β-induced colitis.^191,192^ Based on this, Yue et al. demonstrated that reversing the disrupted intestinal microbial structure can restore fecal metabolic disorders, primarily manifested by an increased abundance of *Lactobacillus* and a decrease in uric acid concentration. Since uric acid is a ligand for the NLRP3 inflammasome, this indirectly inhibits inflammation production.^193^ Similarly, 5-fluorouracil exacerbates mucosal damage by promoting the colonization of *Staphylococcus aureus* and *C. difficile* .^194,195^ Platinum-based drugs and paclitaxel drugs cause widespread inflammatory changes in the colonic mucosa, creating a favorable environment for the overgrowth of *C. difficile* and the production of its exotoxin.^196^

Colitis induced by immune checkpoint inhibitors (ICIs), including anti-CTLA-4 and PD-1/PD-L1 antibodies, exhibits unique immunological characteristics. A clinical meta-analysis revealed that the overall incidence of ICIs-related colitis is 2.4% (95% CI 1.6%–3.6%), with 3–4 severe cases accounting for 1.7%. Combination therapy and anti-CTLA-4 monotherapy pose higher risks.^197,198^ Notably, FMT can reshape the gut microbiota and improve clinical symptoms, suggesting the therapeutic potential of microbial modulation.^199^ Mechanistically, some viewpoints suggest that not only toxic reactions to intestinal tissue but also changes in the gut microbiota caused by ICIs may affect the pathogenesis of colitis. Microbial dysbiosis has been reported as a potential pathophysiology of ICIs-associated colitis.^200,201^ Anti-CTLA-4 treatment in mice leads to a decrease in the abundance of Bacteroidales and Burkholderiales, while supplementation with *Bacteroides fragilis* and *Burkholderia cepacia* can alleviate pathological changes without affecting the antitumor effect.^202^

Microbiome studies have untangled that baseline microbiota characteristics are closely related to the risk of ICIs colitis: individuals enriched with *Firmicutes* (especially *Faecalibacterium*) have a higher incidence.^203^ Furthermore, in existing studies, microbial markers associated with the prevention of CTLA-4 blockade-related colitis have been identified, suggesting that the abundance of the *B. phylum* is closely related to the development of colitis.^204^ Intervention strategies show that *Bifidobacterium* inhibits inflammation by amplifying regulatory T cells, while the tryptophan metabolite indole-3-carbaldehyde maintains epithelial integrity by activating the AhR/IL-22 pathway.^205,206^ These findings highlight the dual role of gut microbiota in anti-tumor drug-associated colitis as both a driver of pathological processes and a potential therapeutic target.

#### Mycophenolate mofetil (MMF)

MMF, a cornerstone immunosuppressant in solid organ transplantation and the treatment of autoimmune diseases, often accompanies significant gastrointestinal toxicity in clinical applications. Epidemiological data reveal that diarrhea (78%), nausea (43%), and abdominal pain (31%) are the most common adverse reactions, while colitis occurs as a severe complication in approximately 9% of cases.^207,208^ Histopathological features exhibit high heterogeneity, with acute colitis (50%), inflammatory bowel disease-like changes (36%), and graft-versus-host disease-like abnormalities (8.3%) being the predominant types. Notably, approximately 47% of suspected cases show negative results on colonoscopy, suggesting the need for random biopsies to enhance diagnostic sensitivity.^209^

The mechanisms underlying MMF’s colonic toxicity involve multi-pathway interactions. First, it selectively inhibits intestinal epithelial cells dependent on de novo purine synthesis (accounting for 50%), leading to decreased mucosal proliferative repair capacity and loss of barrier integrity. Second, MMF induces apoptosis of intraepithelial lymphocytes, weakening local immune surveillance.^139^ Dysbiosis of the gut microbiota plays a critical mediating role in this process.^210^ Animal studies demonstrate that MMF exposure results in the expansion of *Proteobacteria*, predominantly *Escherichia/Shigella* species, while depleting *Akkermansia*, *Parabacteroides*, and *Clostridium*. Germ-free mouse models further confirm that the absence of microbiota can block colonic injury, and broad-spectrum antibiotic intervention can reverse related phenotypes.^211^

Pharmacokinetic studies have untangled that after oral administration, MMF is hydrolyzed to its active form, mycophenolic acid (MPA). Approximately 10% of the glucuronidated metabolite, glucuronidated MPA(MPAG), enters the intestine via the enterohepatic circulation. Intestinal bacteria expressing β-glucuronidase (GUS) remove the glucuronic acid (GA) moiety to generate free MPA and GA, and this metabolic reactivation mediated by GUS is a critical step in the process.^212^ Taylor et al. confirmed through vancomycin intervention experiments that inhibiting GUS-expressing bacteria (such as *Bacteroides spp*. and *Roseburia spp*.) can significantly reduce free MPA levels, thereby alleviating gastrointestinal toxicity.^213,214^ Recent metaproteomic studies further reveal a positive correlation between the abundance of flavin mononucleotide (FMN)-bound GUS protein in the intestines of transplant recipients and the reactivation rate of MPA, providing potential targets for precise intervention.^215^

In terms of clinical management, approximately 98% of MMF-induced colitis cases achieve remission after drug withdrawal, while refractory patients require combination therapy with glucocorticoids or biologics.^216,217^ Given the specificities of long-term immunosuppressive therapy in transplant recipients, complete discontinuation of MMF is often not feasible. Precision modulation strategies based on microbiotaomics exhibit significant clinical value, as they can circumvent the risks of broad-spectrum antibiotics disrupting microbiota homeostasis while effectively managing drug toxicity.

#### Other drug-associated colitis

Proton pump inhibitors (PPIs), as inhibitors of gastric acid secretion, effectively reduce basal and stimulated gastric acid secretion through the irreversible inhibition of the H+/K±ATPase in gastric parietal cells. PPIs have become the first-line treatment for gastroesophageal reflux disease, peptic ulcers, and Zollinger-Ellison syndrome.^218^ Recent studies suggest a potential association between PPI use and the development of colitis.^219^ The pathogenic mechanism may be related to the following microbiome-mediated pathological processes:

(a) Dysbiosis of gut microbiota: Microbial analysis has shown that PPIs users exhibit similar alterations in gut microbiota characteristics as patients with IBD.^220^ PPIs significantly reduce the abundance of anti-inflammatory bacteria (such as *Faecalibacterium*, *Clostridium*, *Turicibacter*, etc.) while promoting the proliferation of proinflammatory bacteria (such as *Streptococcus*, *Ruminococcus*, and *Actinomyces*).^221,222^ It is worth noting that the long-term effects of PPIs on the microbiota structure can even exceed those of antibiotic intervention.^223^ (b) Altered microbiota translocation and colonization: The weakening of the gastric acid barrier promotes the abnormal colonization of microorganisms with colitis-inducing characteristics originating from the oral cavity in the intestine. These microbiota exacerbate inflammatory responses by competing for intestinal mucosal niches.^224^ Additionally, the elevated gastric pH induced by PPIs can significantly enhance the survival of *C. difficile* spores, making it an independent risk factor for this infection.^225,226^

Selective serotonin reuptake inhibitors (SSRIs), as the first-line treatment for depression, have been found to have a significant positive correlation with the risk of microscopic colitis, especially the lymphocytic subtype.^227,228^ This seems to be a result of dysregulation in the neuro–microbiota interaction. SSRIs can specifically inhibit the proliferation of microbiota with biogenic amine transport systems, such as *Lactobacillus spp*., which have a high degree of homology with host neuronal functions.^229^ Animal experiments have confirmed that SSRI intervention leads to an increase in the abundance of *Alistipes*, accompanied by the depletion of beneficial bacteria such as *Bacteroidales S24–7* and *Roseburia*, inhibiting the growth of *Lactobacillus*. This alteration is significantly associated with the colitis phenotype.^230,231^

Furthermore, the diagnosis and management of drug-induced colitis pose challenges. Clinicians need to carefully evaluate patients’ medication history, combined with endoscopic examination and histopathological analysis, to determine the specific type of colitis and its correlation with medications.^190,232^ Currently, this microbiome-mediated protective effect is recognized as colonization resistance, but the specific mechanism of its influence is still poorly understood. Whether microorganisms directly affect pathogens or act on the immune system to affect pathogens remains to be further explored.

### Microscopic colitis (MC)

MC is increasingly recognized as a rare etiology of chronic diarrhea, with chronic watery diarrhea representing its primary clinical manifestation.^233^ Affected patients typically present with 4–9 bowel movements daily, frequently accompanied by abdominal cramps and nocturnal defecation. Notably, endoscopic examinations often fail to reveal macroscopic abnormalities, necessitating histological evaluation for definitive diagnosis through characteristic microscopic changes. The condition demonstrates two principal histological subtypes: lymphocytic colitis (LC), characterized by increased intraepithelial lymphocytes (>20 lymphocytes per 100 epithelial cells vs. normal 3–5), and collagenous colitis (CC), defined by subepithelial collagen band thickening (10–100 μm vs. normal 5–7 μm).^234,235^ A third histological category termed incomplete MC has been proposed, exhibiting borderline collagen thickening (<10 μm) and lymphocyte infiltration (<20 cells), representing histopathological abnormalities that do not fully satisfy diagnostic criteria for LC or CC.^236^

The disease’s cause is yet unknown. The gut microbiota’s dysbiosis has long been thought to be a possible cause. According to the most frequently accepted theory, chronic intestinal inflammation may be primarily caused by pathogenic chemicals found in the luminal material, which may be derived from bacterial flora. Histopathological alterations and symptom relief during fecal diversion, as well as recurrence following intestinal continuity restoration, lend credence to this.^237^ Epidemiological evidence indicates a threefold increased risk of CC development following CDI, persisting for at least 3 y post-infection.^238^ Microbial analyses reveal distinct dysbiosis patterns, with active disease states demonstrating higher microbial dysbiosis indices compared to remission.^239^ Therapeutic interventions with budesonide have been associated with increased α-diversity though β-diversity remains unchanged.^240^ A comprehensive cohort study by Chen et al. identified reduced α-diversity in active microscopic colitis, characterized by the enrichment of pro-inflammatory oral commensals (*Veillonella dispar*, *Haemophilus parainfluenzae*) and depletion of anti-inflammatory species (*Blautia glucerasea, Bacteroides stercoris*).^241^ While *V. dispar*‘s role in intestinal pathology warrants further investigation, its hydrogen sulfide production capacity and *H. parainfluenzae*‘s established association with ulcerative colitis activity suggest potential pathogenic mechanisms.^242^ According to a study by Yannik, anthocyanin extract ACRE might be used to improve the levels of *H. parainfluenzae*, which was found to be considerably positively linked with fecal calprotectin abundance in IBD patients.^243^

Only a few researches, mostly using cross-sectional designs, have looked into particular microbial changes in MC, and their findings have been inconsistent.^239,244^ Positive fecal cultures of *C. concisus* were linked to an increased risk of MC, according to a population-based cohort research conducted in Northern Denmark.^245^ Marta performed biopsies on the mucosa of patients with CC collected via colonoscopy and sequenced their saliva and fecal samples. The results also revealed the significant presence of *C. concisus*. *C. concisus* is an emerging *Campylobacter* species commonly found in the human oral microbiota. This oral-origin pathogen exhibits epithelial barrier-disrupting capabilities in vitro, supported by mucosal biopsy analyses revealing significant colonization in collagenous colitis patients.^246,247^

Concurrently, disease activity correlates with the depletion of protective microbiota, particularly *A. muciniphila* (phylum *Verrucomicrobia*) and butyrate-producing *Alistipes* species.^240,244,248–250^ These microbial alterations may compromise mucin layer integrity and anti-inflammatory capacity, potentially facilitating disease progression.

Notably, microscopic colitis demonstrates distinct clinical and microbiological features compared to IBD, exhibiting milder symptomatology and more stable microbial profiles.^244^ Intriguingly, the fecal microbiome of CC patients in remission resembles healthy controls while sharing certain dysbiotic features with IBD, suggesting potential transitional states along the IBD spectrum. This observation supports the conceptualization of microscopic colitis as a “pre-IBD” condition, where microbial investigations may yield insights for IBD prevention strategies. Current research remains limited by the predominance of cross-sectional studies and inconsistent findings regarding specific microbial alterations. Elucidation of microbiota-pathogenesis relationships in microscopic colitis could therefore provide novel therapeutic targets for both colitis management and IBD prevention.

## Infectious colitis

### Campylobacter jejuni infectious colitis

*C. jejuni*, a spiral-shaped, *gram-negative microaerophilic bacterium*, is the second leading cause of gastroenteritis in the US, with over 2.4 million annual cases (including unreported sporadic infections).^251^ In the last decade, *C. jejuni* has been one of the most zoonotic pathogens worldwide, with 120,000 human infections reported in the European Union in 2020.^252^ Understanding its virulence factors and pathogenesis mechanisms is crucial for combating C. jejuni-induced colitis.

Through transcriptomic profiling of 192 clinical strains, Kovács et al. identified oxidative stress resistance, biofilm formation, and metabolic adaptation as key survival strategies.^253^ In IL-10^−/−^ mice, *C. jejuni* infection induced severe enterocolitis resembling human campylobacteriosis,^254^ triggering mixed Type1/Type17 cytokine responses where IL-17 suppressed bacterial survival in gut epithelia.^255^ Notably, IFN-γ+ ILCs (exhibiting ILC3-to-ILC1 plasticity) drovet T cell-independent colitis,^256^ while PI3K-AKT and MAPK pathways emerged as key pathogenic mediators.^257–259^

Proteomic analysis by Jiang et al. confirmed PI3K-AKT activation in *C. jejuni* colitis,^258^ which is consistent with its downstream mTOR/NF-κB signaling. FMT ameliorated infection by enriching *Akkermansia* and metabolites (butyrate/deoxycholic acid) that inhibit PI3K-AKT,^259–261^ effects replicated by oral metabolite administration. This highlights FMT’s dual mechanism correcting dysbiosis while targeting pathogenic signaling. The PI3K-AKT-mTOR-NF-κB axis was functionally validated in IL-10^−/−^ NF-κB-EGFP mice showing enhanced colitis with bacterial dissemination and elevated CXCL2/IL-17α/IL-1β.^258^ Rapamycin (mTOR inhibitor) reduced bacterial persistence and inflammation while enhancing splenic clearance. Parallel studies confirmed TLR/NF-κB’s role in colitis progression, positioning this signaling network as promising therapeutic targets.

### Salmonella enterica serovar Typhimurium infectious colitis

*Salmonella enterica serovar Typhimurium* (*S. Typhimurium*), a gram-negative facultative intracellular pathogen, is a major cause of human infectious colitis. Murine models using antibiotic-pretreated mice recapitulate key disease features by enhancing intestinal colonization.^262^ Mechanistically, impaired PPARγ expression, LACC1 deficiency, elevated ASB1 expression, and TAB2 stabilization drive colitis pathogenesi,^262,263^ with gut dysbiosis, hypoxia, and host-microbe imbalance emerging as critical contributing factors.

Probiotic supplementation (*Lactobacillus*, *Akkermansia*, *Bifidobacterium longum*, etc.) effectively mitigates colitis by restoring microbial diversity.^264–268^ Notably, *L. acidophilus* ameliorates characteristic of crypt hyperplasia, goblet cell depletion, and MUC2 downregulation via Notch signaling modulation.^265^ Synergistic strategies demonstrate enhanced efficacy: combined *B. longum* and active vitamin D3 (VD3) suppress inflammation while boosting antimicrobial peptide production,^266^ whereas VD3-butryate co-administration reinforces intestinal barrier function through AhR-mediated tight junction regulation.^269^ Crucially, butyrate-induced ZBP-89 orchestrates Tph1/5HT axis activation during *S. Typhimurium* infection.^270,271^

Intestinal inflammation exerts dual effects on pathogen dynamics: while initially promoting *S. Typhimurium* overgrowth through microbiota disruption, it subsequently reduces luminal pathogen load by 10^5^-fold post-infection.^272^ This paradox, coupled with nutrient availability’s impact on probiotic populations, underscores the complex interplay shaping colitis progression.

### Escherichia coli infectious colitis

As a regular inhabitant of the gut microbiota throughout human life, *E.coli* plays a controversial role in colitis. Some strains, including *E. coli Nissle 1917* (*EcN*) and *E. coli strain CEC15 (CEC)*, function as intestinal probiotics, which are beneficial for the treatment of infectious colitis,^273^ while *adherent invasive E. coli* (*AIEC*) tend to aggravate colitis.^274,275^

*E. coli LF82*, an *AIEC* strain, has been linked to dysbiosis and colitis pathogenesis.^25^ In an acidic environment within macrophages, RstAB is activated and promotes the transcription of csgD and asr in *E. coli LF82*, causing biofilm formation and acid tolerance, eventually contributing to the replication of *E. coli LF82* within macrophages and intestinal colonization.^276^ Under *E. coli LF82* infection, the Th17 and Tregs differentiation balance was severely affected. By regulating the Th17/Tregs balance and damaging the intestinal flora composition, *E. coli LF82* infection can damage the intestinal mucosal barrier and aggravate intestinal inflammation in colitis.^277^ *E. coli* adhesion protein, FimH, is another virulence factor in *E. coli* infectious colitis. Via upregulation of CD11b+CD103-dendritic cell activities in the mature LNs, FimH induces Th1 and Th17 differentiation, thus breaking the immune balance of the host, which aggravates the disease progression of *E. coli* infectious colitis.^278^ Notably, during infection, IL-17 may promote IL-22 secretion and play a protective role in *E. coli* infectious colitis.^279^

*Enterohemorrhagic E. coli* (*EHEC*) is another strain of the human pathogenic *E. coli* bacteria that can cause serious colitis.^280,281^ *L. casei* is one probiotic that showed both prevention and treatment effects on colitis induced by *EHEC strain O157:H7*, which provided sights for exploring therapies to reduce *E. coli* infection by probiotics.^282^

*EcN* is a probiotic bacterium that is beneficial for the recovery of infectious colitis. Dacquay identified 187 differentially expressed genes in the colon tissues under *EcN* infection, among which genes involved in flagella biosynthesis and motility, as well as the formate hydrogenlyase complex, emerged as potential key transcriptional responses against inflammation.^283^ Meanwhile, *EcN* could produce a high concentration of a free LCFAs hydroxylated on the third carbon (LCFA-3OH): C18-3OH, which served as an agonist of the PPARγ. Via regulating microbiota–host interactions, C18-3OH of *EcN* alleviated colitis progression, further supporting the therapeutic role of *EcN* in colitis and nominating the C18-3OH as the functional factor of *EcN* .^284^ On the other hand, *E. coli* may promote the recovery of colitis in mice via activation of the TLR4/NF‑κB signaling pathway. Commensal *CEC* is another probiotic strain. In a chronic colitis model, both *EcN* and *CEC* significantly decreased the severity of colitis, with increased gene expression involved in gut homeostasis under inflammatory settings and downregulated myeloperoxidase activity and CD3+ immune-cell infiltration. However, compared to *EcN*, *CEC* has a better beneficial impact on the colitis.^285^

In cooperation with other drugs, *EcN* may exert enhanced function in alleviating colitis. Minocycline is a tetracycline with immunoregulatory properties.^286^ *EcN* supplementation in combination with minocycline treatment could effectively improve the recovery process of the intestinal damage and prevent the reoccurrence of colitis.^287^ Mechanistically, downregulated expression of TNF-α, IL-1β, IL-2, iNOS, and MMP-9, together with increased expression of MUC-3 and ZO-1, restored the microbiota composition induced by colitis-altered microbiota composition.^287^

### Infectious colitis induced by other pathogens

**Figure 3.**
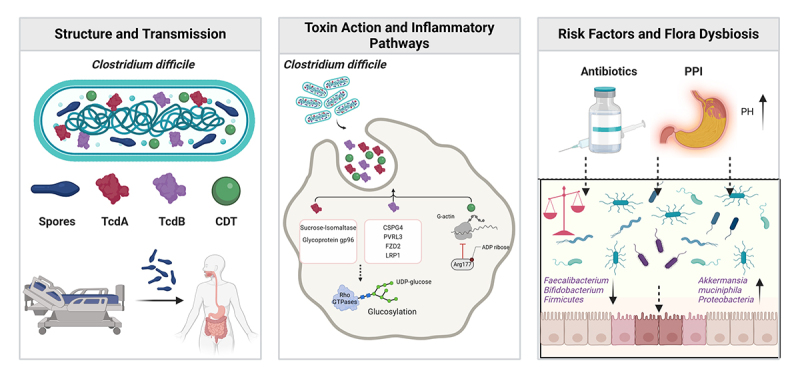
Pathogens including *Campylobacter jejuni*, *Salmonella enterica serovar typhimurium Escherichia coli* and *Shigella*, etc are displayed. Potential mechanism of infectious colitis induced by different pathogens are summarized accordingly.

*Shigellae*, a member of the *Enterobacteriaceae* family, can cause a range of symptoms, from minor diarrhea to potentially fatal dysentery.^288^ *Shigellae* abundance after treatment has been extensively examined for colitis recovery as a measure of therapeutic efficacy.^289–292^ Infectious colitis has also been linked to pathogens such as *Helicobacter hepaticus*, *COVID-19*, *Mycobacterium Gordonae*, and *C. difficile* (Figure 3). ^293–296^

## Special types of colitis

### Eosinophilic colitis (EC)

EC is characterized by a significant infiltration of eosinophils in the intestinal wall, and its diagnosis requires the simultaneous presence of symptoms of colonic dysfunction and histological evidence of segmental or diffuse eosinophilia.^297^ It is worth noting that a similar eosinophil increase may also be observed in mucosal biopsies of patients with IBD.^298^ However, EC can be distinguished from IBD through the presence of more abundant co-localized deposits of IgE and tryptase.^299^ Due to the absence of specific biomarkers, the diagnosis of EC still relies on exclusion, necessitating the sequential elimination of other secondary factors that cause colonic eosinophilia, such as drug reactions and parasitic infections.^300^ This diagnostic dilemma further hinders the elucidation of the pathophysiology of EC.

### Immune regulatory mechanisms of eosinophils in EC

Eosinophils are regionally distributed in the colonic mucosa, with the highest concentration found in the ascending colon of both adults and children.^301,302^ These cells respond to various stimuli, including nonspecific tissue damage, allergens, and infections. Their recruitment is jointly regulated by the activation of Th2 cells, cytokines such as IL-5/IL-13, and the chemokine eotaxin.^303,304^ Activated eosinophils release four major cationic proteins through degranulation: eosinophil peroxidase (EPO), eosinophil cationic protein (ECP), eosinophil-derived neurotoxin (EDN), and major basic protein (MBP). Among these, MBP can disrupt the intestinal epithelial barrier function by downregulating occludin expression.^305^ It is noteworthy that there are differences in the immune mechanisms of EC between infants and adults: the former is often associated with IgE-mediated mast cell accumulation and degranulation, while the latter is primarily a non-IgE-related disease dominated by CD4^+^ Th2 lymphocytes.^306^

### Potential connection between microbiota and the pathogenesis of EC

The hygiene hypothesis suggests that reduced microbial exposure due to modern hygienic conditions may be linked to increased incidence of allergies and autoimmune diseases.^307^ The decline in gut microbiota diversity caused by western lifestyle factors (such as a high-fat diet and antibiotic overuse) may promote the development of EC through Th2 immune imbalance.^308^ Studies have demonstrated that certain allergic and autoimmune diseases can be prevented by infection with specific bacteria, viruses, or parasitic pathogens.^309,310^ The interaction within the microbiota-eosinophil immune axis explains the rapid activation of eosinophils during microbiota dysbiosis.^311^ The chronic course of the disease may persist through two mechanisms: 1) gut microbiota imbalance triggered by occult intestinal infections and 2) abnormal immune responses of the host to commensal microbiota.^312^

The establishment of gut microbiota during infancy is crucial for immune homeostasis, and appropriate microbial stimulation can correct the balance of the Th2 immune response. Clinical studies indicate a reduction in *Bifidobacteria* and an enrichment of *Clostridium spp*. in the guts of allergic infants, along with the increased abundance of conditional pathogens like *E. coli* and *S. aureus* .^313,314^ Comparative studies between urban and rural children further confirm that the high microbial diversity in rural environments can reduce allergy risks by modulating the abundance of *Bacteroides* and *Ruminiclostridium spp*. .^315^

Experimental research reveals that *R. gnavus* can penetrate the intestinal mucus layer, stimulating epithelial secretion of IL-13, increasing IgE production and mast cell trypsin expression. This activates the ILC2-eosinophil axis and induces Th2 differentiation and cytokine production, ultimately leading to eosinophil infiltration in tissues.^316^ *Gram-negative bacteria* (e.g., *H. influenzae*, *E. coli*) can induce eosinophils to release ECP and MBP, while *Gram-positive bacteria* (e.g., *S. aureus*) primarily trigger the release of EPO.^317^ It is worth noting that *B. longum* can reduce colonic EPO levels by inhibiting Th2 differentiation,^318^ indicating that microbiota composition directly affects eosinophil function.

Mucosal iNKT cells participate in eosinophil regulation through the overexpression of CXCL16, Vα24, and CD1d, and their abnormal accumulation can exacerbate colitis.^319^ Importantly, gut microbiota influences iNKT cell homeostasis by modulating CXCL16 expression: mucosal iNKT cells in germ-free mice proliferate excessively, while early microbial colonization can block this pathological process.^320^ Recent studies have untangled that *F. rodentium* promotes intestinal epithelial renewal (increased Ki67+ crypt cells) by reducing the proportion of eosinophils while simultaneously reshaping immune homeostasis.^321^ This suggests that microbiota not only directly regulates immune cell function but also affects eosinophil activity through indirect pathways like epithelial barrier repair, providing a multifaceted perspective on the pathophysiology of EC.

Current research suggests that chronic inflammation in EC may be maintained by occult intestinal infections or abnormal immune responses to the gut microbiota.^312^ Further studies are needed to determine the impact of microbial dysbiosis on the clinical outcomes of eosinophilic colitis and to explore the symbiosis between the microbiota and eosinophils. Specific research areas include: (a) the causal relationship between specific bacterial species and the Th2 signaling pathway; (b) the regulation of eosinophil function by microbial metabolites (such as secondary bile acids); and (c) the clinical efficacy of targeted microbiota interventions (such as FMT and probiotics). By analyzing the microbe-epithelium-immune interaction network, it is hoped that new diagnostic and therapeutic strategies can be developed for EC.

### Clostridioides difficile infection (CDI) related colitis

*C. difficile* is a *Gram-positive anaerobic spore-forming bacterium* whose spores can spread via the fecal-oral route. This bacterium exhibits strong environmental tolerance (resistant to UV light, heat, and antibiotics) in the medical setting.^322,323^ Initially isolated from neonatal feces, it has now become the primary pathogen causing iatrogenic diarrhea. Its clinical manifestations range from asymptomatic carriage and mild diarrhea to life-threatening pseudomembranous colitis.^324,325^ Spore formation is driven by a sigma factor cascade reaction regulated by Spo0A, leading to asymmetric cell division and spore generation.^326^ When the gut microbiota is disrupted due to factors such as antibiotic use, the spores germinate into toxin-producing vegetative cells, establishing dominant colonization through mechanisms such as consuming SCFAs and inhibiting SBA production.^327^

#### Risk factors: from antibiotics to microbial dysbiosis

The onset of CDI is often preceded by the exposure to certain risk factors that disrupt the intestinal microbiota, providing an opportunity for *C. difficile* to colonize, proliferate, and produce toxins,^328^ ultimately leading to the development of colitis. Antibiotic exposure stands as the most central risk factor for CDI, particularly with specific antibiotics such as clindamycin, fluoroquinolones, and cephalosporins posing a higher risk of *C. difficile* exposure compared to other antibiotics, while tetracycline demonstrates a relatively lower inductive risk.^329,330^ It is worth noting that PPIs increase the risk of CDI by altering the gut microbiota structure through the reduction of gastric acid pH although there remains controversy regarding their discontinuation during treatment.^331^ The combination or prolonged use of multiple antibiotics further exacerbates microbial dysbiosis, thereby elevating the risk of CDI.^332,333^

#### Toxin networks: from cellular destruction to immunestorms

The pathogenicity of CDI is primarily mediated by Toxin A (TcdA) and Toxin B (TcdB), both encoded by the virulence gene cluster (TcdR, TcdE, TcdL, and TcdC) and belonging to the Large Clostridial Toxin (LCT) family.^334^ Functioning as glucosyltransferases, TcdA/B inactivate host cell Rho GTPases through glycosylation, subsequently disrupting the actin cytoskeleton, triggering cell cycle arrest, cytokine secretion, and intestinal epithelial necrosis.^335^ TcdA specifically binds to sucrase-isomaltase and glycoprotein gp96, while TcdB exhibits a more complex receptor spectrum, including chondroitin sulfate proteoglycan-4 (CSPG4), poliovirus receptor-like 3 (PVRL3), members of the Wnt receptor frizzled (FZD) family (namely FZD2), and low-density lipoprotein receptor-related protein 1 (LRP1).^336^ Following receptor binding, the toxins enter cells via endocytosis, although the endocytic pathway remains incompletely understood. Recent studies have revealed that TcdB escapes degradation by inhibiting lysosome activity, thereby enhancing its intracellular toxicity.^337^ Once inside the cytoplasm, TcdA/B utilizes UDP-glucose to catalyze the glycosylation of Rho GTPases, disrupting the GTPase cycle of Rho proteins (members of the Ras superfamily) and impeding critical processes such as cell migration, phagocytosis, and apoptosis.^338,339^ The glycosylation of Rac1 by TcdB inhibits cyclin D1, blocks the G1-S phase transition in epithelial cells, and significantly delays mucosal repair.^340^ Additionally, TcdA/B activates the apoptosis-associated speck-like protein (ASC)-dependent inflammasome, leading to the massive release of IL-1β and exacerbating intestinal inflammatory responses.^341^

In addition to TcdA/B, the role of binary toxin (CDT) in CDI has garnered increasing attention. CDT consists of ADP-ribosyltransferase (ADPRT), and its catalytic subunit, CDTa, modifies the G-actin Arg177 site, thereby inhibiting actin polymerization and triggering cytoskeletal depolymerization.^342,343^ However, the toxicity of CDT is highly dependent on the presence of TcdA/B: in a hamster model lacking TcdA/B, CDT alone only causes atypical intestinal bleeding.^344^ It’s worth noting that CDT is closely associated with the pathogenicity of hypervirulent strains (such as RT027). In mouse models, CDT induces IL-1β secretion through the TLR2/NF-κB pathway and promotes eosinophil apoptosis, exacerbating intestinal damage.^345^ Clinical studies further untangle that patients infected with CDT^+^ strains exhibit higher incidence rates of severe illness, complications, and recurrence, and their mortality rate is eight times higher than that of CDT^−^ patients.^346,347^ This hypervirulence may be associated with CDT^+^ strains carrying multiple drug resistance genes (such as tetracycline and moxifloxacin resistance genes), which enhance their colonization advantage through horizontal transfer.^348^

In summary, *C. difficile* toxins synergistically disrupt intestinal homeostasis through multifaceted mechanisms: TcdA/B directly damages the epithelial barrier and triggers an inflammatory storm, while CDT amplifies pathological effects by regulating the cytoskeleton and immune response. The combined action of these three factors leads to loss of colonic epithelial integrity, delayed repair, and systemic inflammatory responses, ultimately resulting in typical CDI phenotypes such as diarrhea and pseudomembrane formation.^349,350^

#### Dysbiosis: from symbiotic collapse to ecological hijacking

The pathogenesis of CDI not only relies on the direct damage caused by toxins but is also closely associated with the disruption of intestinal microbiota homeostasis. CDI patients commonly exhibit a significant reduction in gut microbial diversity, characterized by the depletion of protective microbiota and abnormal proliferation of opportunistic pathogens.^351^ Specifically, there is a decrease in the abundance of butyrate-producing *Faecalibacterium*, *Bifidobacterium*, as well as *Ruminococcaceae* and *Lachnospiraceae* within the *Firmicutes*, leading to reduced synthesis of SCFAs and subsequent impairment of intestinal epithelial energy metabolism and barrier function.^352,353^ Additionally, *C. scindens*, which plays a role in bile acid metabolism, is reduced in CDI mouse models, and its absence hinders the conversion of primary bile acids to SBA, further promoting the germination of *C. difficile* spores.^354^

It is noteworthy that the abundance of *A. muciniphila* is abnormally elevated in CDI patients, and its metabolites may provide a carbon source for *C. difficile*, forming a “metabolic symbiosis” relationship.^355^ Concurrently, there is an increase in the abundance of members of the *Proteobacteria*.^356^ This microbial dysbiosis triggers the collapse of inflammation-repair homeostasis, ultimately leading to intestinal barrier breakdown and systemic inflammatory responses.

**Figure 4.**
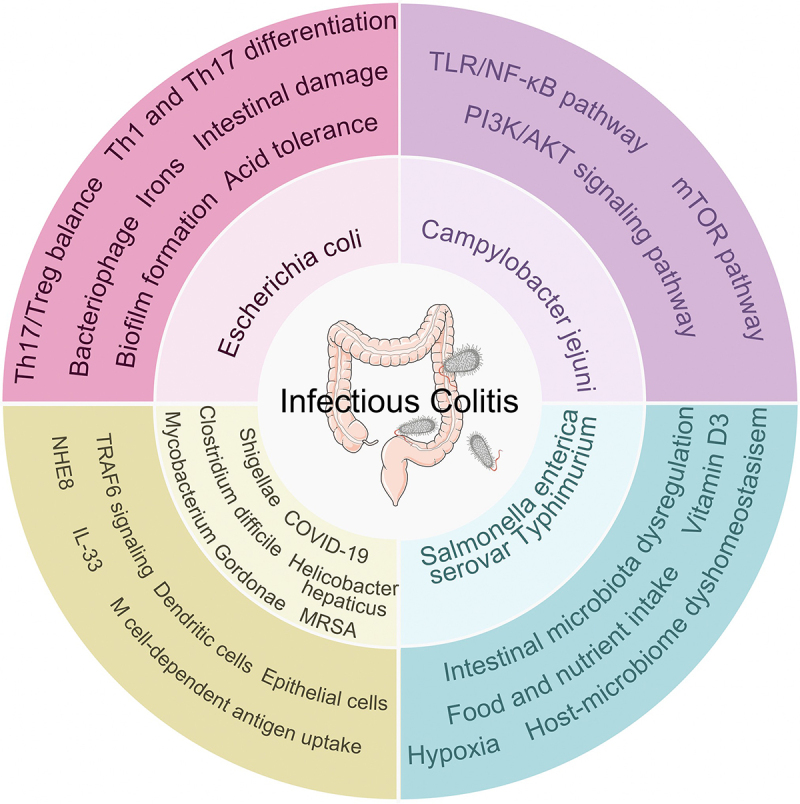
Pathogenic pathways and risk factors for CDI-infected colitis.

*C. difficile* infection remains a significant challenge in nosocomial infections, and its increasing risk of recurrence demands attention from clinicians. Besides addressing risk factor exposure and exercising caution in the use of antibiotics and PPIs, the application of FMT from healthy patients and bezlotoxumab has shown promising benefits.^357–359^ These interventions provide insights into the role of microbiota in CDI pathogenesis, shifting the focus to gut microbes. Overall, as our understanding of *C. difficile* grows, recognizing the crucial role of microorganisms in the gut offers a unique perspective on CDI and opens up new possibilities for its treatment (Figure 4).

## Treatment strategies for colitis

**Figure 5.**
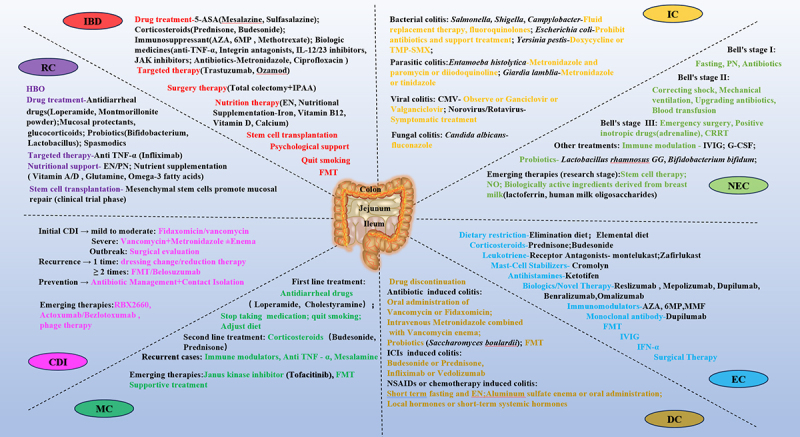
Treatment strategies for colitis.

The treatment of colitis involves a multifaceted approach tailored to the type, severity, and individual patient needs. First-line strategies typically include anti-inflammatory medications such as aminosalicylates (e.g., mesalamine) or corticosteroids to reduce acute inflammation. For moderate-to-severe cases, immunosuppressants (e.g., azathioprine) or biologic therapies (e.g., anti-TNF agents like infliximab) target specific immune pathways to control symptoms and maintain remission. Antibiotics may be prescribed for infectious colitis or complications like abscesses. Lifestyle modifications, such as adopting a low-residue or anti-inflammatory diet, avoiding trigger foods, and managing stress, complement medical therapies. In refractory cases, surgery – such as colectomy for ulcerative colitis – may be necessary. Emerging options like JAK inhibitors (e.g., tofacitinib) and investigational approaches like FMT show promise. Supportive care, including hydration, pain management, and regular monitoring through imaging or colonoscopy, ensures comprehensive management. A personalized, multidisciplinary plan involving gastroenterologists, dietitians, and surgeons is crucial to optimizing outcomes and quality of life (Figure 5).

## Conclusion

All things considered, we believe that disruption of the gut microbiota plays a major role in the development of different types of colitis. Intestinal inflammation is the result of a sequence of “butterfly effects” caused by the imbalances in the gut microbiome caused by the interaction of the gut microbiota with elements such the host immune system, environment, and genetics. Our findings imply that investigating non-pharmacological therapeutic options requires a thorough understanding of gut flora. In fact, significant advancements in treatment approaches that target the microbiome have been made throughout the last 10 y. For example, FMT has been explored as a possible therapeutic option in clinical settings to help patients with immune-mediated disorders linked to dysbiosis regain healthy microbiome configurations, such as when treating an infection caused by *C. difficile*. However, there are several difficulties because the “healthy configuration” of the human microbiome is dynamic and complicated, going beyond simple microbial replacement. All things considered, the creation of these microbiome-based treatments necessitates a better understanding of the relationships between the microbiome and the intestinal milieu of the host, which calls for additional etiological study.

Most current studies are cross-sectional analyses, making it difficult to reveal the dynamic process of colitis development. It is necessary to conduct long-term cohort studies to track the entire progression of patients from early inflammation to chronicity or carcinogenesis, particularly focusing on the temporal interaction networks of gut microbiota, immune cell subsets, and metabolites. Existing biomarkers lack sufficient specificity, and there is a need to identify more predictive indicators. Additionally, deep learning-based classification models for colitis severity have achieved high accuracy. If combined with biomarkers such as fecal volatile organic compounds or serum, multimodal diagnostic tools can be constructed to enhance the precision of early identification and prognostic assessment. The role of gut microbiota in colitis has been extensively validated. Future research should focus on developing personalized therapies based on microbiota typing, such as customized probiotics/prebiotics: designing microbiota modulation strategies tailored to specific enterotypes. In addition, colitis can be diagnosed and treated through interventions targeting metabolic products, nano-delivery systems, and the integration of interdisciplinary technologies. In summary, future studies should concentrate on elucidating dynamic mechanisms, screening precise biomarkers, and implementing multi-dimensional intervention strategies, thereby advancing colitis diagnosis and treatment toward a personalized and preventive paradigm.

## Data Availability

The authors confirm that all data underlying the findings are fully available without restriction.

## Abbreviation

5ASA: 5 aminosalicylic acid;
6MP: 6-Mercaptopurine;
AZA: Azathioprine;
CMV: Cytomegalovirus;
CRRT: continuous renal replacement therapy;
EN: Enteral nutrition;
G-CSF:HBO: Recombinant human granulocyte colony-stimulating factor; Recombinant human granulocyte colony-stimulating factor;
ICIs: Immune checkpoint inhibitors;
IFN-α: Interferon alpha;
IL-12/23: Interleukin-12/23;
IPAA: Ileal pouch anal anastomosis;
IVIG: Intravenous immunoglobulin;
NO: Nitric oxide;
TMP-SMX: trimethoprim/sulfamethoxazole;
